# Supplementary material for: Dietary Regulation of Oxidative Stress in Chronic Metabolic Diseases
Source: Foods. 2021 Aug 11;10(8):1854. doi: 10.3390/foods10081854 (PMC8391153; doi:10.3390/foods10081854)
Supplement: Supplementary file 1 [file foods-10-01854-s001.zip › foods-1335872-supplementary.pdf]

Dietary regulation of oxidative stress in chronic metabolic diseases

Shuai Jiang [1], Hui Liu [1] and Chunbao Li [1,*]

Table S1 Chronic inflammatory diseases in Western diet population and Mediterranean diet population

Dietary pattern	Experimental objects	Methods and Assessment	Major finding	Reference
Western diet	2284 participants	A community-based prospective study (4.7 years)	Western dietary was significantly increased the pattern changes in serum insulin for CVD	Mirmiran et al. [51]
	50 healthy adult participants	A cross-sectional study with food frequency questionnaire	An obesogenic diet may sensitize healthy individuals to acute stress on cognitive performance	Delgado et al. [52]
	44444 men (26 years)	A prospective cohort study with food frequency questionnaire	A higher Western dietary pattern score was associated with an increased risk for gout	Rai et al. [53]
	255 cohort (60–64 years)	A food frequency questionnaire	Western dietary was each independently associated with smaller left hippocampal volume and hippocampal atrophy	Felice et al. [54]
	6204 women (50-69 years)	A cross-sectional study	Western dietary pattern has the highest correlation with chronic diseases	Markussen et al. [55]
	Older adults in China	A randomized experiment	Western diet was significantly related to obesity, hypertension and the metabolic syndrome	Jing et al. [56]
	5267 children in China	A multistage random	Western diet was significantly associated with higher concentrations of low-density lipoprotein cholesterol, triglycerides and a lower high-density lipoprotein cholesterol	Shang et al. [57]
Mediterranean diet	93,122 women (50–79 years)	A WHI study (10.5 years)	Mediterranean dietary may be associated with a lower risk of cardiac sudden death	Bertoia et al. [69]
	7769 participants (20–65 years)	A doetinchem cohort study (10 years)	Mediterranean diet was associated with a 23% lower risk of composite CVD	Hoevenaar-Blom et al. [70]

4172412 subjects	A Meta-analyses studies	Mediterranean diet was an 8% reduction of overall mortality, a 10% reduced risk of CVD and a 4% reduction of neoplastic disease	Sofi et al. [71]
7447 persons (55 to 80 years)	A multicenter trial (4.8 years)	Mediterranean diet supplemented with extra-virgin olive oil or nuts reduced the incidence of cardiovascular events in high cardiovascular risk	Estruch et al. [72]
235 subjects (66.5 years)	A randomized, parallel-design, controlled trial	Mediterranean diet supplemented with extra-virgin olive oil or nuts reduced 24-hour ambulatory BP, total cholesterol, and fasting glucose in high cardiovascular risk	Domenech et al. [73]
1424 adult	A food frequency questionnaire (10 years)	Higher Mediterranean diet is associated with better cognitive performance, therefore less cognitive decline in older but not middle-aged individuals	Wade et al. [74]
2,258 subjects (65 and over)	A longitudinal with dietary composite questionnaire (4.5 years)	Higher Mediterranean diet was associated with a reduced risk of developing and progression to AD	Scarmeas et al. [75]
2,247 subjects (65 and over)	A longitudinal with the Godin leisure time exercise questionnaire (5.4 years)	Higher Mediterranean diet adherence was associated with reduced risk for AD	Scarmeas et al. [76]
110 females (55 to 80 years)	A large trial (1 years)	Mediterranean diet can reduce the contents of urinary F2-IP and urinary 8-oxo-dG, and reduce the oxidative damage to lipids and DNA in Metabolic syndrome individuals	Mitjavila et al. [77]
